# Supplementary material for: A Wide-Range-Response Piezoresistive–Capacitive Dual-Sensing Breathable Sensor with Spherical-Shell Network of MWCNTs for Motion Detection and Language Assistance
Source: Nanomaterials (Basel). 2023 Feb 24;13(5):843. doi: 10.3390/nano13050843 (PMC10005261; doi:10.3390/nano13050843)
Supplement: Supplementary file 1 [file nanomaterials-13-00843-s001.zip › nanomaterials-2192531-supplementary.pdf]

Support information

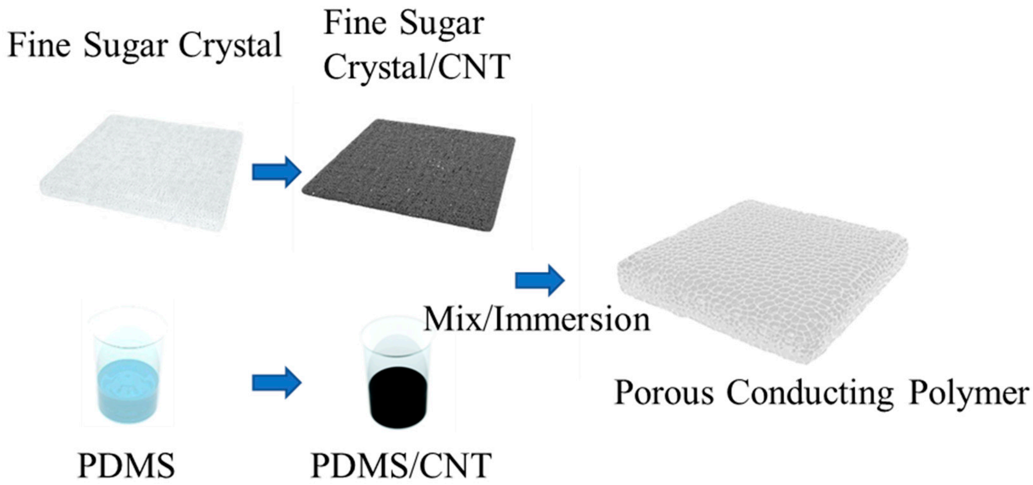

Figure S1. Sensor preparation process.

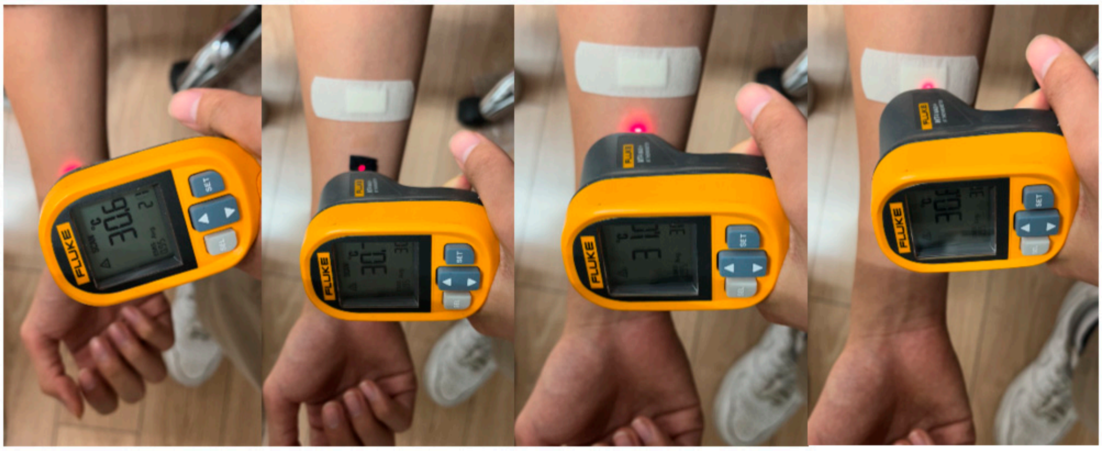

Figure S2. Arm skin temperature was 30.6℃ before the material was placed, 30.7℃ after the porous structure strain sensor was placed for 3 hours, 31.0℃ after the PDMS was placed for 3 hours, and 30.8℃ after the Band-aid was placed for 3 hours.

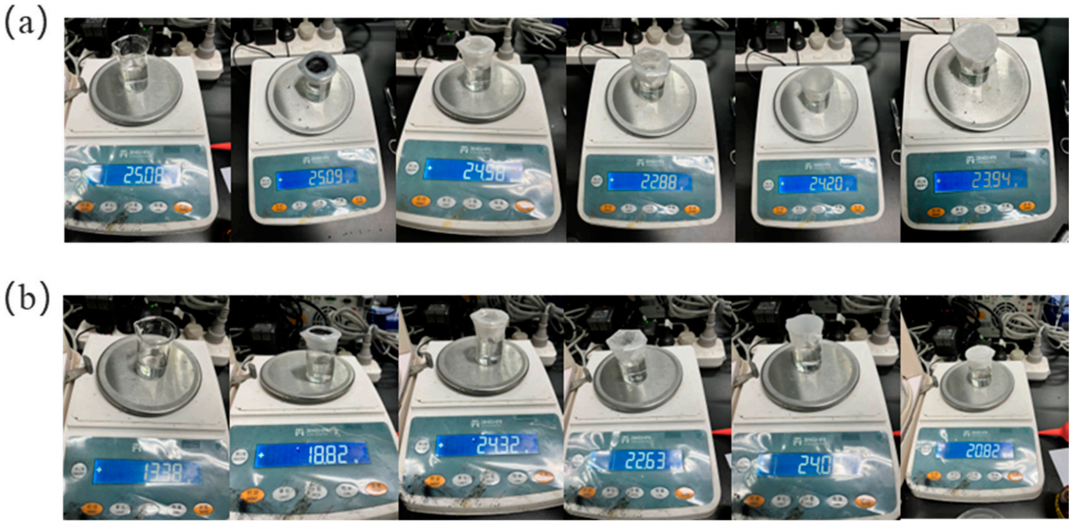

Figure S3. The air permeability test used six sets of materials sealed with water beakers placed at room temperature to record water evaporation from (a) day 0 to (b) day 15.

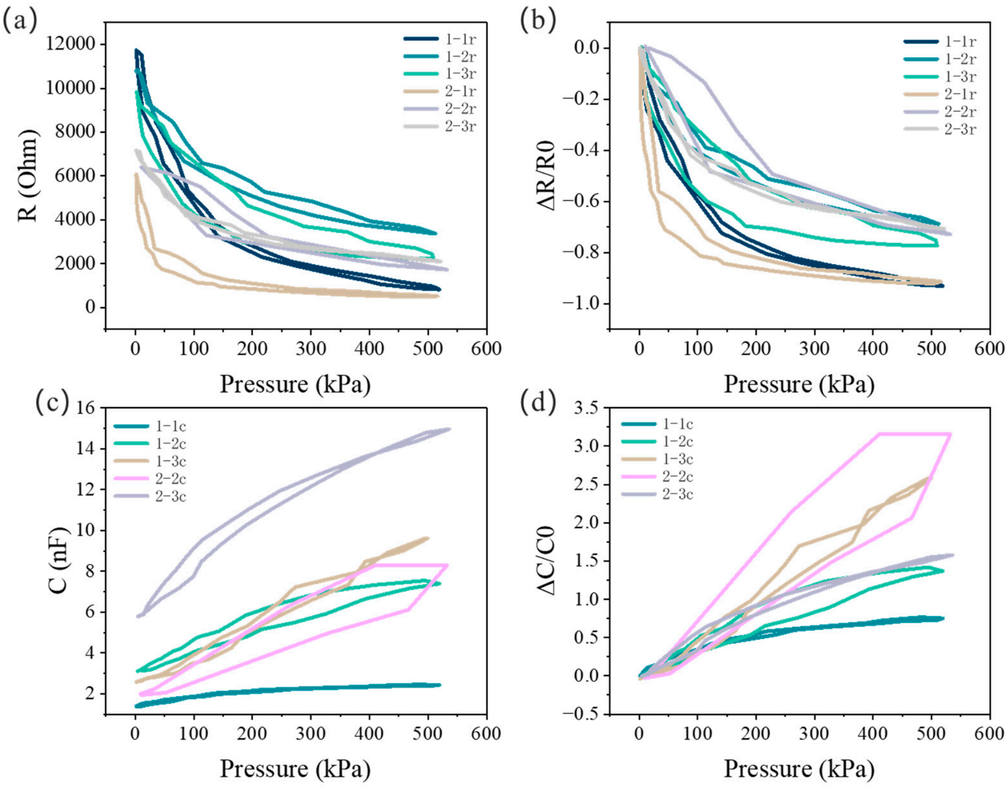

Figure S4. The (a) resistance, (b) resistance sensitivity, (c) capacitance and (d) capacitance sensitivity of the sensor under pressure cycling were tested when the mass ratio of MWCNTs in the PDMS matrix was different from that on the surface of the porous structure.

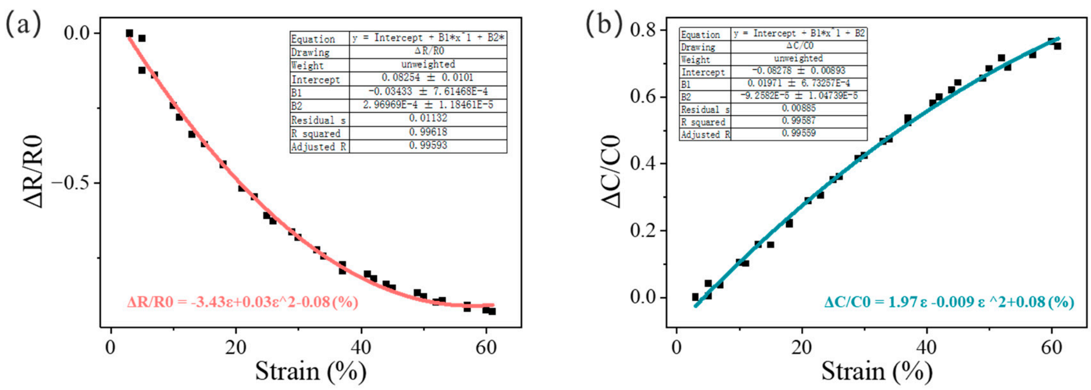

Figure S5. Equation fitting of (a) ΔR/R0 and (b) ΔC/C0 properties of sensors

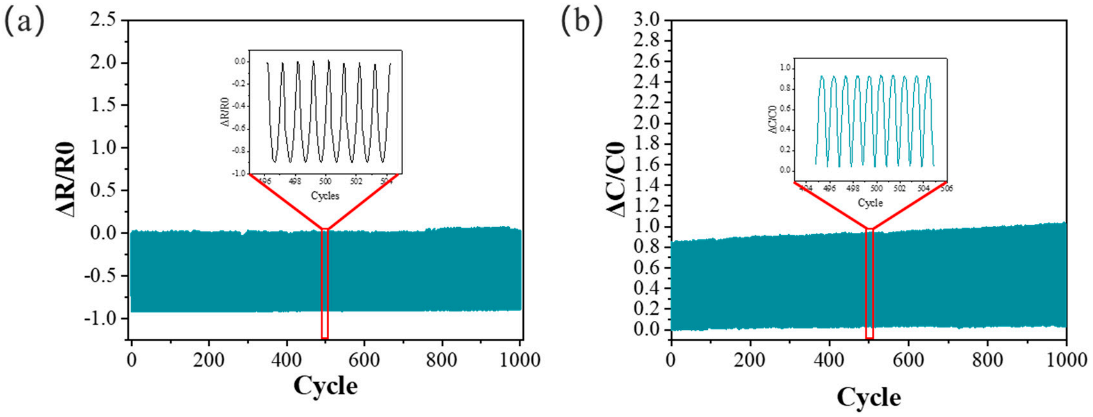

Figure S6. The response curve of sensor (a) resistance and (b) capacitance under 1000 compression cycles and its local magnification diagram.

Table S1. Summary of the performance of the porous stress sensors.

Materials	Sensing mechanism	Detection range	Sensitivity	Cycles	Reference
CNTs/TPU	Piezoresistive	0.7 Pa-160 kPa	1.02 kPa^{-1}	6000	[55]
CNT/TPU	Piezoresistive	0-200 kPa	0.032 kPa^{-1}	-	[56]
MWCNTs-rGO@PU	Piezoresistive	0-50 kPa	0.088 kPa^{-1}	5000	[57]
hierarchically porous PDMS	Capacitive	0-400 kPa	0.18 kPa^{-1}	10000	[58]
PDMS@CNCs	Piezoresistive	0-450 kPa	0.0082 kPa^{-1}	2000	[59]
ZnO/PDMS	Capacitive	0-50 kPa	0.717 kPa^{-1}	4000	[60]
CB/PDMS	Piezoresistive	0-500 kPa	0.0048 kPa^{-1}	500	[61]
PDMS/CNT	Piezoresistive	6 Pa-50 kPa	0.9 kPa^{-1}	255	[62]

PDMS/MWCNTs/NaCl	Piezoresistive	1 Pa-100 kPa	10.805 kPa^{-1}	1000	[63]
CNT-PDMS	Piezoresistive<br>Capacitive	1 kPa-520 kPa	1.2 kPa^{-1}<br>0.38 kPa^{-1}	1000	Our work

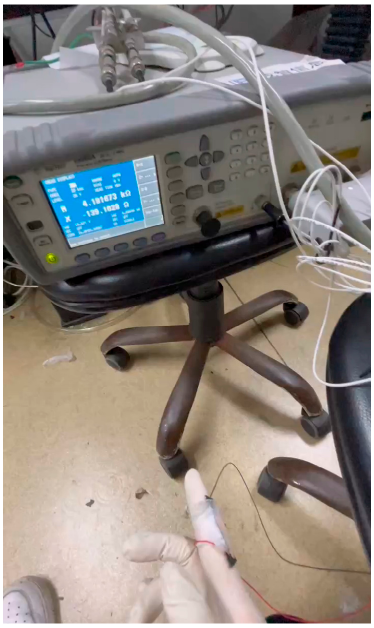

Movie S1. Finger motion detection

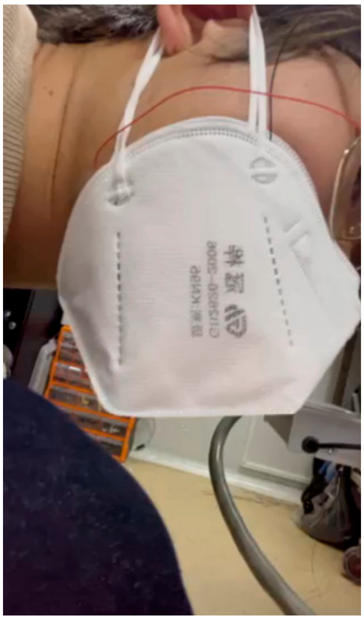

Movie S2. Speech detection

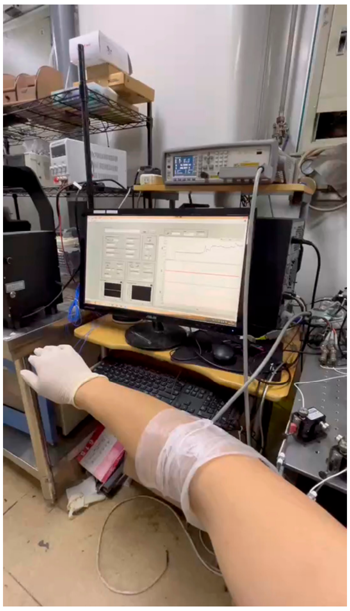

Movie S3. Arm motion detection

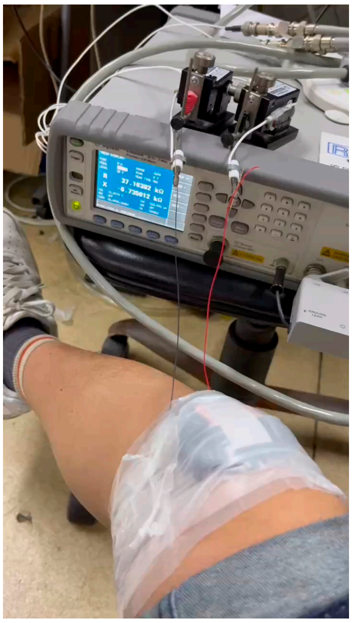

Movie S4. Leg motion detection

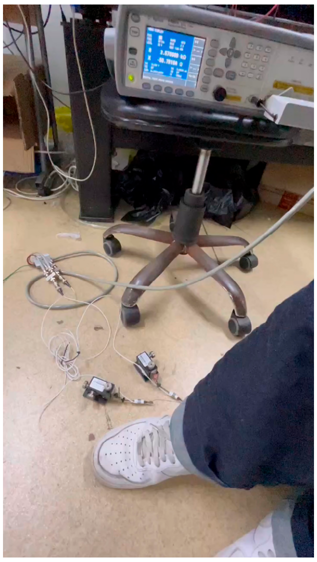

Movie S5. Foot motion detection

Reference

[55] Yin, Y.M., et al., *Facile Fabrication of Flexible Pressure Sensor with Programmable Lattice Structure.* ACS Applied Materials & Interfaces, 2021. **13**(8): p. 10388-10396.

[56] Lee, J., et al., *Ultra-robust wide-range pressure sensor with fast response based on polyurethane foam doubly coated with conformal silicone rubber and CNT/TPU nanocomposites islands.* Composites Part B: Engineering, 2019. **177**.

[57] Tewari, A., et al., *Highly Exfoliated MWNT–rGO Ink-Wrapped Polyurethane Foam for Piezoresistive Pressure Sensor Applications.* ACS Applied Materials & Interfaces, 2018. **10**(6): p. 5185-5195.

[58] Hwang, J., et al., *Fabrication of hierarchically porous structured PDMS composites and their application as a flexible capacitive pressure sensor.* Composites Part B: Engineering, 2021. **211**.

[59] Mu, C., et al., *Flexible strain/pressure sensor with good sensitivity and broad detection range by coupling PDMS and carbon nanocapsules.* Journal of Alloys and Compounds, 2022. **918**.

[60] Hsieh, G.-W., L.-C. Shih, and P.-Y. Chen, *Porous Polydimethylsiloxane Elastomer Hybrid with Zinc Oxide Nanowire for Wearable, Wide-Range, and Low Detection Limit Capacitive Pressure Sensor.* Nanomaterials, 2022. **12**(2): p. 256.

[61] Zhu, G., et al., *3D Printed Skin-Inspired Flexible Pressure Sensor with Gradient Porous Structure for Tunable High Sensitivity and Wide Linearity Range.* Advanced Materials Technologies, 2022. **7**(7): p. 2101239.

[62] Iglio, R., et al., *Flexible Polydimethylsiloxane Foams Decorated with Multiwalled Carbon Nanotubes Enable Unprecedented Detection of Ultralow Strain and Pressure Coupled with a Large Working Range.* ACS Applied Materials & Interfaces, 2018. **10**(16): p. 13877-13885.

[63] Li, W., et al., *Synergy of Porous Structure and Microstructure in Piezoresistive Material for*

High-Performance and Flexible Pressure Sensors. ACS Applied Materials & Interfaces, 2021. **13**(16): p. 19211-19220.
